# Phase Modulation in Rydberg Dressed Multi-Wave Mixing processes

**DOI:** 10.1038/srep10462

**Published:** 2015-06-08

**Authors:** Zhaoyang Zhang, Huaibin Zheng, Xin Yao, Yaling Tian, Junling Che, Xiuxiu Wang, Dayu Zhu, Yanpeng Zhang, Min Xiao

**Affiliations:** 1 Key Laboratory for Physical Electronics and Devices of the Ministry of Education & Shaanxi Key Lab of Information Photonic Technique, Xi’an Jiaotong University, Xi’an 710049, China; 2 Department of Physics, University of Arkansas, Fayetteville, Arkansas 72701, USA & National Laboratory of Solid State Microstructures and Department of Physics, Nanjing University, Nanjing 210093, China

## Abstract

We study the enhancement and suppression of different multi-waving mixing (MWM) processes in a Rydberg-EIT rubidium vapor system both theoretically and experimentally. The nonlinear dispersion property of hot rubidium atoms is modulated by the Rydberg-Rydberg interaction, which can result in a nonlinear phase shift of the relative phase between dark and bright states. Such Rydberg-induced nonlinear phase shift can be quantitatively estimated by the lineshape asymmetry in the enhancedand suppressed MWM processes, which can also demonstrate the cooperative atom-light interaction caused by Rydberg blockaded regime. Current study on phase shift is applicable to phase-sensitive detection and the study of strong Rydberg-Rydberg interaction.

The phase modulation as well as the refractive index modification in a Rydberg medium, caused by electric fields produced either externally or internally owing to the interparticle interactions, is of central importance in nonlinear optics, laser technology, quantum optics and optical communications^1^. Because the high-lying Rydberg electron is very far from the core of the atom, the atom possesses exaggerated properties, such as huge polarizability that scales as *n*^7^, where *n* is the principle quantum number. These properties lead to strong and tunable Rydberg-Rydberg interactions^2^,^3^,^4^ among the atoms, which can render the Rydberg medium intrinsically nonlinear. For example, Rydberg electromagnetically induced transparency (EIT) makes the transmission through the medium highly sensitive to electric fields^1^, which can enable modifications on the refractive index and nonlinear phase shift due to the interparticle interactions in the nonlinear processes associated with EIT.

Comparing with the other nonlinear optical processes, the multi-waving mixing (MWM) processes in Rydberg-EIT medium have unique features^5^,^6^,^7^,^8^, and one typical feature is that the coherence time of the generated signal is shorter than the time of ionization^9^, while it is known that the incoherence plasma formation in Rydberg gases is ~100 *ns* or longer^10^,^11^. With EIT configuration, the coherence between the ground state and highly-excited Rydberg states is well established, which can enhance the efficiency of the MWM processes^12^,^13^. In addition, the spatial arrangement of EIT configuration will suppress the Doppler width greatly, which makes atoms in the beam volume behave like cold atoms with reduced Doppler effect^14^,^15^,^16^,^17^. Finally, the EIT windows will pick up the corresponding MWM signals with narrow linewidth (less than 30 MHz). Therefore, probing the EIT-assisted MWM processes can provide a powerful spectral method to investigate the properties of Rydberg atoms.

In this paper, we study the enhancement and suppression of Rydberg dressed MWM processes with the assistance of EIT windows in a hot Rb atomic system both theoretically and experimentally. The enhanced and suppressed MWM signals are significantly modified via the relative phase control^18^ due to the nonlinear dispersion property modification induced by corresponding dressing effects and the cooperative nonlinear effect^19^,^20^ from the Rydberg blockade regime. The introducing of strong Rydberg-Rydberg interactions into atom-light interaction means that each atom can no longer be treated independently and the correlations between atoms must be taken into consideration, which can be interpreted as a cooperative effect. The cooperative nonlinearity in an atomic ensemble can be much more obvious for high atomic density. As s result, the spatial effects of corresponding dressed signals can visually advocate the change of nonlinear dispersion property in current experiment. The intensity evolutions of enhancement and suppression results may map onto the nonlinear phase shift in modulated dispersion property by scanning dressing fields. Different from the asymmetry degree of cavity transmission profile method^21^, such nonlinear phase shift with background-free advantages can be estimated via the dressing asymmetry in enhanced and suppressed MWM linshapes, which can also demonstrate the excitation blockade effects. The nonlinear phase shift of the relative phase between dark and bright states gives a novel way for studying the Rydberg-Rydberg interactions and phase-sensitive detection.

## Results

**Figure 1:**
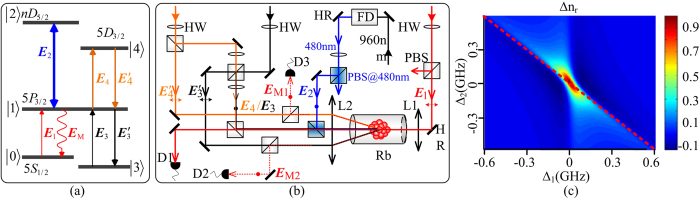
(**a**) A five-level atomic system in Rydberg-EIT rubidium atom for dressed MWM processes. (**b**) Experimental setup for different MWM processes. L-lens, D-detector, FD-frequency doubler, HW-half wave plate with corresponding wavelength, PBS-polarized beam splitter with corresponding wavelength. Double-headed arrows and filled dots denote horizontal polarization and vertical polarization of the incident beams, respectively. (**c**) Theoretical calculations corresponding to the change in refractive index (Δ*n*_r_) of a medium for a probe laser (or MWM signal) frequency versus Δ_1_ and Δ_2_. Ω_1_ = 2π × 54 MHz, Ω_2_ = 2π × 7.6 MHz, Ω_4_ = 2π × 142 MHz, Ω_4_′ = 2π × 224 MHz. The atom density is 1.0 × 10^12^ cm^−3^.

An X-type five-level ^85^Rb atomic system, consisting of two hyperfine states F = 3 (|0〉) and F = 2 (|3〉) of the ground state 5S_1/2_, a first excited state 5P_3/2_ (|1〉), a lower-lying excited state 5D_3/2_ (|4〉), and a highly-excited Rydberg state *n*D_5/2_ (|2〉), is used to generate the EIT-assisted MWM processes. Six laser beams derived from four commercial external cavity diode laser systems with frequency-stabilized servos are coupled into the corresponding transitions as shown in Fig. 1(a). The experimental setup is shown in Fig. 1(b). Except for the ***E***_4_′, the experimental setup is essentially the same as previous work^22^. A weak laser beam ***E***_1_ (780.24 nm with a diameter of 0.8 mm, frequency *ω*_1_, wavevector ***k***_1_) from LD1 probes the lower transition |0〉 to |1〉, while a pair of coupling beams ***E***_3_ (780.23 nm, *ω*_3_, ***k***_3_) and ***E***_3_′ (*ω*_3_, ***k***_3_′), derived from the same LD3 with a small angle between them both with the same diameter of 1 mm, connect another lower transition |3〉 to |1〉. To excite hot rubidium atoms from level |1〉 to Rydberg states |2〉, we obtain the needed 480 nm laser ***E***_2_ (*ω*_2_, ***k***_2_) by the way of frequency doubling LD2 at ≈960 nm. The strong beam ***E***_2_ (diameter 1 mm) adding onto the beam ***E***_3_ (in the same direction), which counter-propagates with beam ***E***_1_, drives the highly-excited Rydberg transition |1〉 to |2〉. ***E***_4_ (775.98 nm with a diameter of 1 mm, frequency *ω*_4_, wavevector ***k***_4_) and ***E***_4_′ (*ω*_4_, ***k***_4_′) from LD4 drive the transition |1〉 to |4〉.

Different-order dressed MWM processes can be obtained by turning the incident beams on selectively. First, by blocking beams ***E***_3_ and ***E***_3_′, a four-wave mixing (FWM) process ***E***_FWM1_ with the phase-matching condition (PMC) ***k***_FWM1_ = ***k***_1_ + ***k***_4_ − ***k***_4_′ can be dressed by ***E***_2_ in the Y-type four-level subsystem |0〉↔|1〉↔|2〉↔|4〉. Next, when opening all other beams except ***E***_4_′, a non-EIT-assisted FWM process ***E***_FWM2_ (with ***k***_FWM2_ = ***k***_1_ + ***k***_3_ − ***k***_3_′ in the Λ-type three-level subsystem |0〉↔|1〉↔|3〉) and two EIT-assisted six-wave mixing (SWM) proesses^14^,^23^,^24^ ***E***_SWM1_ involving in Rydberg states and ***E***_SWM2_ (with the PMCs of ***k***_SWM1_ = ***k***_1_ + ***k***_3_ − ***k***_3_′ + ***k***_2_ − ***k***_2_ and ***k***_SWM2_ = ***k***_1_ + ***k***_3_ − ***k***_3_′ + ***k***_4_ − ***k***_4_) can be observed in |0〉↔|1〉↔|3〉↔|2〉 and |0〉↔|1〉↔|3〉↔|4〉, respectively. These MWM signals have the same emitting direction (opposite to the direction of ***E***_3_′, as shown in Fig. 1(b)) except for ***E***_FWM1_ (propagating along the opposite direction of ***E***_4_′). The various MWM processes are identified by tuning the frequency detuning of corresponding coupling beams and detected by respective avalanche photodiode detectors (APD). Specifically, the MWM processes related to the Rydberg state |2〉 may be called Rydberg MWM signals with strong Rydberg-Rydberg interactions.

The interaction among Rydberg atoms scales with *n*^11^ and leads to the change in refractive index of the medium and nonlinear phase shift of the relative phase between dark and bright states, which can be mapped onto the enhancement and suppression of EIT-assisted MWM processes with dressing effects. To be specific, the modification of refractive index (*n*_r_) caused by Rydberg energy level shift^22^ (Δ*ω*_2_) can be expressed as


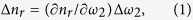


where *∂n*_r_/*∂ω*_2_ = (*n*_g_ − 1)/*ω*_2_, *ω*_2_ is the Rydberg state coupling laser frequency and *n*_g_ is the group refractive index. The theoretical simulation of Δ*n*_r_ is shown in Fig. 1(c). The phase modulation (ΔΦ_1_) due to the strong cooperative atom-light interaction due to Rydberg blockade is described as


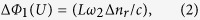


which means the phase shift is proportional to the Rydberg induced dispersion change Δ*n*_r_ and the propagation distance *L* (or equivalently atomic density). See Methods for the theoretical derivations of Δ*n*_r_ and ΔΦ_1_(*U*).

For the two FWM signals (via the pathways 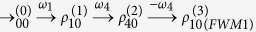 and 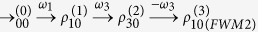) with the dressing effects of ***E***_2_ and ***E***_4_, the corresponding third-order polarizations *P*^(3)^ for the output FWM signals under steady-state condition are given by


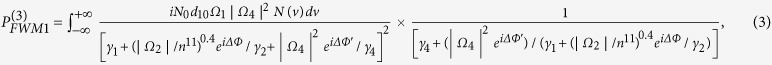


,


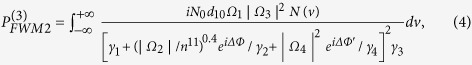


,where *N*(*v*) = *N*_*0*_exp(−*v*^2^/*u*^2^)/*uπ*^1/2^ is the particle number density in terms of speed distribution function^15^; Ω_*i*_ = *d*_*ij*_*E*_*ij*_/*ħ* (*i,j* = 1, 2…) is the Rabi frequency between |*i*〉´|*j*〉, and *d*_*ij*_ is the dipole momentum; *N*_0_ is the atom density; *γ*_1_ = (Γ_10_ + Γ_*t*_) + *i*(Δ_1_ + *k*_1_*v*), *γ*_2_ = (Γ_20_ + Γ_*c*_ + Γ_*t*_) + *i*(Δ_1_ + Δ_2_) + *i*(*k*_1_ − *k*_2_)*v*, *γ*_3_ = (Γ_30_ + Γ_*t*_) + *i*(Δ_1_ + Δ_3_) + *i*(*k*_1_-*k*_3_)*v*, *γ*_4_ = (Γ_40_ + Γ_*t*_) + *i*(Δ_1_ + Δ_4_) + *i*(*k*_1_-*k*_4_)*v*; Γ_*ij*_ = (Γ_*i*_ + Γ_*j*_)/2 is the decoherence rate between |*i*〉 and |*j*〉; Γ_*i*_ is the transverse relaxation rate determined by the longitudinal relaxation time and the reversible transverse relaxation time; Δ_*i*_ = *ω*_*ij*_ − *ω*_*i*_ is the detuning between the resonant transition frequency *ω*_*ij*_ and the laser frequency *ω*_*i*_ of ***E***_*i*_. Note that the collision ionization rate Γ_*c*_^25^, transit time Γ_*t*_ and the Doppler effect (*kv*) should be considered. For the two EIT-assisted SWM signals via 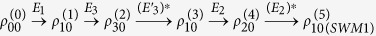 and 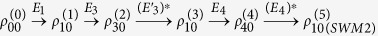, the corresponding fifth-order polarizations *P*^(5)^ are given by


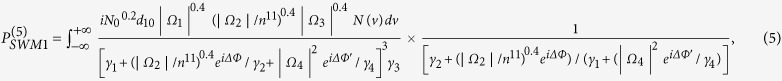


,


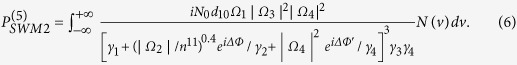


.

Here, the additional phase factors *e*^*i*ΔΦ^ and *e*^*i*ΔΦ′^ are introduced into the dressing terms (|Ω_2_|/*n*^11^)^0.4^/*γ*_2_ and |Ω_4_|^2^/*γ*_4_ to account for the propagation effect. ΔΦ = ΔΦ_1_ + ΔΦ_2_, where ΔΦ_1_(*U*) is the phase modulation induced by the possibly coherent Rydberg-Rydberg interaction *U*; the relative phase ΔΦ_2_ and ΔΦ′ are related to the orientations of induced dipole moments and can be manipulated^18^ by corresponding laser frequency detuning and Rabi frequency.

### Phase modulated intensity and spatial effects in the Y-type subsystem

**Figure 2:**
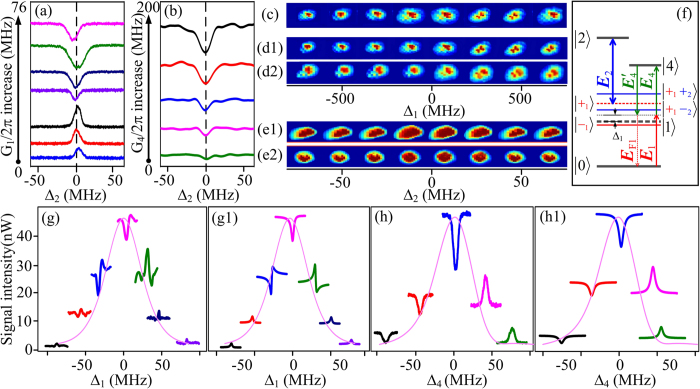
Dressed FWM1 process by scanning the frequency of Rydberg state (37D) coupling field ***E***_2_. (**a**) Switching between enhanced peak to suppressed dip by increasing Ω_1_ at Δ_1_ = −Δ_4_ = 30 MHz. (**b**) Dependence of suppressed dip on Ω_4_ at Δ_1_ = Δ_4_ = 0. (**c**) The probe field images versus Δ_1_. (d1- d2) The ***E***_4_ EIT images without/with ***E***_2_ EIT dressing versus Δ_1_ at discrete points of Δ_2_ = Δ_4_ = −Δ_1_. (e1-e2) The dressed ***E***_2_ EIT and FWM1 images versus Δ_2_ with Δ_1_ = Δ_4_ = 0. (f) Dressed energy level configurations with ***E***_2_ and ***E***_1_ dressing. (**g-h**) are the evolutions of dressed FWM1 versus Δ_2_ by tuning Δ_1_ at Δ_4_ = 0 and tuning Δ_4_ at Δ_1_ = 0, respectively. (**g1**) and (**h1**) are corresponding theoretical predictions for (**g**) and (**h**) The Lorentzian profiles are the FWM1 signals versus Δ_1_ and versus Δ_4_. Ω_1_ = 2π × 54 MHz at 0.5 mW, Ω_2_ = 2π × 7.6 MHz at 200 mW, Ω_4_ = 2π × 142 MHz at 6 mW, Ω_4_′ = 2π × 224 MHz at 15 mW. The atom density is 1.0 × 10^12^ cm^−3^.

Figure 2 shows the dressed FWM1 process in the Y-type four-level subsystem |0〉↔|1〉↔|2〉↔|4〉 by scanning the frequency of Rydberg coupling field ***E***_2_. Suppressed and enhanced FWM1 signals (the suppressed condition is Δ_1_ + Δ_4_ = 0 and the enhanced condition is 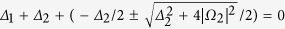 are observed by changing the frequency detuning of ***E***_1_ or ***E***_4_. According to the new two-photon dressed rule^26^, the moving states |±〉 will impose influence on the enhancing and suppressing results of MWM signals. Let’s first show the generating process of Rydberg-dressing enhancement and suppression simply. Figure 2(a) shows the switch from an enhanced peak to a suppressed dip by growing Ω_1_ at Δ_1_ = −Δ_4_ = 30 MHz. The dressing processes can be considered as following: first, as shown in Fig. 2(f), level |1〉 is split into the dressed states |±_1_〉 by ***E***_1_; and then |+_1_〉 is split into |+_1_±_2_〉 secondly by ***E***_2_. Therefore, once the dressing level |+_1_〉 moved around the position of Δ_1_, the suppressed condition is satisfied and the suppressed case of FWM1 occurs in Fig. 2(a). Figure 2(b) shows the dependence of suppressed dip on the strength of ***E***_4_ at Δ_1_ = Δ_4_ = 0. The enhanced condition cannot be satisfied in the situation shown in Fig. 2(b) in which the suppressed dip increases as the power of ***E***_4_ increases and the two-step dressing process can be simplified as level |1〉 is split into |±_1_〉.

In order to visually investigate the nonlinear dispersion property induced by Rydberg dressing effect and cooperative effect, we turn to the spatial effects on the images of dressed signals. With only ***E***_1_ and ***E***_2_ turned on, Fig. 2(c) shows the focusing/defocusing effects of probe signal versus Δ_1_. Nonlinear refractive index *n*_*r*_ is negative in the self-focusing medium (Δ_1_ < 0) while positive in the self-defocusing one (Δ_1_ > 0). Figure 2(d1,d2) show the probe images with ***E***_1_&***E***_4_ and ***E***_1_&***E***_2_&***E***_4_ on versus Δ_1_, respectively. With ***E***_2_ blocked, the focusing/defocusing effects of probe images at different Δ_1_ + Δ_4_ = 0 can be stronger than the effects in Fig. 2(c) due to the growing of absolute value of refractive index. With ***E***_1_&***E***_2_&***E***_4_ on, the images of dressed ***E***_4_ EIT become more defocusing compared with the corresponding ones in Fig. 2(d1) due to Δ*n*_*r*_ is negative in most part of the resonance line as shown in Fig. 1(c). In addition, the spatial splitting and shift in Fig. 2(d2) can be attributed to 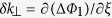, where ΔΦ_1_ can be modified as


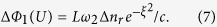


Figure 2(e1,e2) are the images of dressed ***E***_2_ EIT and Rydberg dressed FWM1 versus Δ_2_, respectively. The dressed FWM1 and dressed ***E***_2_ EIT with Δ_1_ = Δ_2_ = Δ_4_ = 0 are much more defocusing than the points of Δ_2_≠0. All the signal images visually advocate the modulation on dispersion property due to the existence of Rydberg-Rydberg interaction.

Figure 2(g) shows the change in dressed enhancement and suppression of FWM1 by increasing the frequency detuning Δ_1_ at Δ_4_ = 0. The Lorentzian profile (curve constituted of the baseline of each signal) is a one-photon peak of the FWM1 signal versus Δ_1_ and can be described by the single-photon term *γ*_1_ in Eq. (3). The intensity of FWM1 in Fig. 2(g) is first suppressed and then enhanced at Δ_1_ = −32 MHz, while it is first enhanced and then suppressed at Δ_1_ = 32 MHz. Obviously, a dressing asymmetry occurs with Δ_1_ = 0 considered as a center.

In general, the dressing enhancement peaks and suppression dips are symmetrical distributed along the center. However, the induced nonlinear phase shift may lead to the asymmetry^18^,^21^ in the lineshapes of dressed MWM signals. To estimate such dressing asymmetry quantitatively, we define the asymmetry factor as


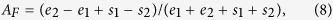


where *e*_*i*_ and *s*_*i*_ represent the enhancement and suppression of FWM1 intensity; subscripts 2 and 1 indicate *e*_*i*_ (or *s*_*i*_) are taken with Δ_1_ > 0 and Δ_1_ < 0, respectively. Actually, the relationship between A_F_ and phase shift can be described as


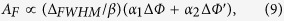


where Δ_FWHM_ and β are the full width at half maximum (FWHM) and full width at a certain frequency detuning point of the corresponding profile, respectively; α_1_ and α_2_ are the ratio parameters for phase shift ΔΦ and ΔΦ′caused by ***E***_2_ and ***E***_4_, respectively.

According to Eq. (8), the value of A_F_ in Fig. 2(g) is about 0.58 at |Δ_1_| = 32 MHz. Due to the absence of Autler-Townes (AT) splitting on the profile, the dressing effect of ***E***_4_ on the one-photon term *γ*_1_ that only affects the intensities of the signals can be neglected. Since the modulated results of FWM1 in Fig. 2(g) are related to the change in Δ_1_, one can attribute the results to the dressing effect of ***E***_2_ on *γ*_1_. Therefore, A_F_ in Fig. 2(g) is mainly contributed by the Rydberg dressing and cooperative nonlinear effect. The denominator of Eq. (3) is simplified to [*γ*_1_ + (|Ω_2_|^2^/*n*^11^)^0.4^*e*^iΔΦ^/*γ*_2_]^2^*γ*_4_ and can explain Fig. 2(g) well by setting ΔΦ = ΔΦ_1_(*U*) + ΔΦ_2_ = −π/3. (see Fig. 2(g1)).

Figure 2(h) is the modulated enhancement and suppression of FWM1 signal by increasing Δ_4_ at Δ_1_ = 0, and A_F_ is about 0.91 at |Δ_4_| = 50 MHz. Different from the case in Fig. 2(a), the Lorentzian profile (curve constituted of the baseline of each signal) is a two-photon peak of the FWM1 signal versus Δ_4_, which can be described by the two-photon term *γ*_4_ in Eq. (3). Obviously, the change of Δ_4_ can also affect the modulated results of FWM1, and it can be ascribed to the dressing effect of ***E***_2_ on *γ*_4_ associating with self-dressing shown in Eq. (3). As a consequence, the denominator of Eq. (3) is simplified as 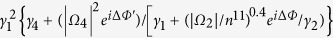, which can account for Fig. 2(h) with ΔΦ = ΔΦ_1_(*U*) + ΔΦ_2_ = −π/3 and ΔΦ′ = −π (see Fig. 2(h1)).

### Phase modulated intensity in the inverted-Y type subsystem

**Figure 3:**
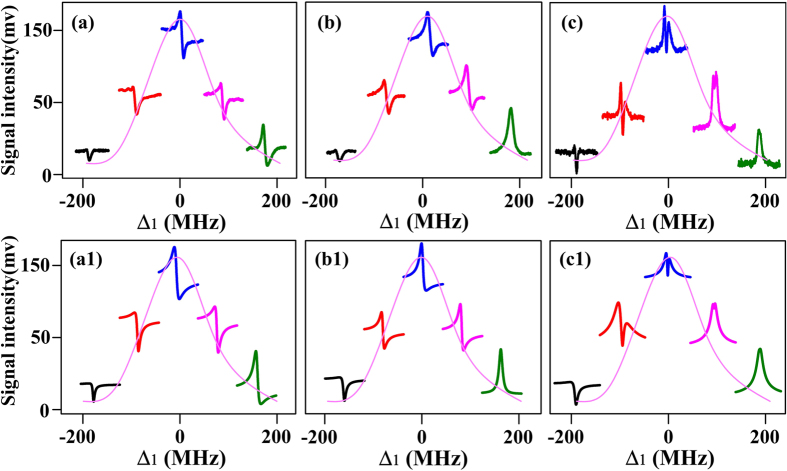
The change in Δ_1_ induced enhancement and suppression of FWM2 together with the SWM2 process by scanning Δ_4_ (**a**) without ***E***_2_, and (**b**) with ***E***_2_ coupling the transition between 5P_3/2_↔54D_5/2_, respectively, at atom density *N*_0_ = 1 × 10^12^ cm^−3^. (**c**) is the same to (**b**) except for *N*_0_ = 2.4 × 10^12^ cm^−3^. The profile (curve constituted of the baseline of each signal) in each panel is the FWM2 signal versus Δ_1_, which is broadened by the Doppler effect Δ_1_−Δ_3_ = *k*_1_*v* + *k*_3_*v*. (a1)(b1) and (c1) are the theoretical predictions corresponding to (**a**)(**b**) and (**c**), respectively. (a1) ΔΦ′ = −*π*/6. (b1) ΔΦ′ = −*π*/6, ΔΦ = ΔΦ_1_ + ΔΦ_2_ = −*π*/12. (c1) ΔΦ′ = −*π*/6, ΔΦ = ΔΦ_1_ + ΔΦ_2_ = −*π*/3. Δ_2_ = 0, Δ_3_ = 150 MHz. Ω_1_ = 2π × 54 MHz at 0.5 mW, Ω_2_ = 2π × 7.6 MHz at 200 mW, Ω_4_ = 2π × 116 MHz at 4 mW, Ω_3_ = 2π × 170 MHz at 5 mW, Ω_3_′ = 2π × 275 MHz at 13 mW.

Now, we try to pick out the phase shift induced by the Rydberg blockade. Figure 3 shows the enhanced and suppressed FWM2 coexisting with the SWM2 by scanning Δ_4_ at discrete Δ_1_. To be specific, Fig. 3(a) is the case with ***E***_2_ beam blocked and shows the dressing effect of ***E***_4_ on FWM2 versus Δ_4_ at different Δ_1_, which can be well simulated by Eq. (4) by setting ΔΦ′ = −*π*/6 at Δ_3_ = 150 MHz (see Fig. 3(a1)). As defined above, the dressing asymmetry factor A_F_ in Fig. 3(a) is 0.19 at |Δ_1_| = 80 MHz. The profile (curve constituted of the baseline of each signal) in Fig. 3(a) is the one-photon peak of FWM2 signal versus Δ_1_ (see the one-photon term *γ*_1_ in Eq. (4)) with ***E***_2_ blocked, and the peak is broadened to be 200 MHz by the Doppler effect Δ_1_−Δ_3_ = *k*_1_*v* + *k*_3_*v*. Figures 3(b,c) are the ones with the dressing effect of ***E***_2_ (coupling the transition between 5P_3/2_↔54D_5/2_) at different atomic densities, respectively. The profiles in Figs.3 (b) and (c) are the peaks of FWM2 signal together with SWM2 signal by scanning Δ_1_. However, the dressed FWM2 signal is restrained in a narrower range by the EIT configuration of |0〉↔|1〉↔|4〉. Compared with Fig.3(a), A_F_ values in Fig. 3(b,c) increase to be as high as 0.61 and 0.86 at |Δ_1_| = 80 MHz due to the introducing of Rydberg field. The difference between the asymmetry factors on the profiles can be explained by the nonlinear phase shift caused by ***E***_2_ dressing effect and the cooperative atom-light interaction^27^. Since both Fig. 3(b,c) are related to the same Rydberg state 54D_5/2_, the phase shift induced by the change of cooperative nonlinearity due to Rydberg-Rydberg interaction can be observed by comparing the modulated results of *N*_0_ = 1 × 10^12^ cm^−3^ and *N*_0_ = 2.4 × 10^12^ cm^−3^. The introducing of correlations between atoms into atom-light interaction can lead to a cooperative effect. The increase of Rydberg atom population will increase the cooperative nonlinearity and result in a dramatically change of the measured lineshapes.

Comparing the fourth curve in Fig. 3(b) with the fourth one at Δ_1_ = 80 MHz in Fig. 3(a), the difference between the modulated results can be explained well by setting ΔΦ = ΔΦ_1_ + ΔΦ_2_ = −*π*/12 (see Fig. 3(b1)). For the higher density shown in Fig. 3(c), the theoretical prediction agrees well with the experimental results by setting ΔΦ = ΔΦ_1_ + ΔΦ_2_  = −*π*/3 (see Fig. 3(c1)). Obviously, the phase shift as well as the dressing asymmetry factor grows with the atomic density and such density-dependent characteristic can demonstrate the ΔΦ_1_ caused by the change of cooperative nonlinearity. Considering that the values of ΔΦ_2_ in Fig. 3(b,c) are almost same due to the saturated dressing effect, the phase difference caused by the increase of cooperative nonlinear effect is approximately *π*/4. Therefore, such results sufficiently prove the existence of the phase shift induced by the interaction between Rydberg atoms.

**Figure 4:**
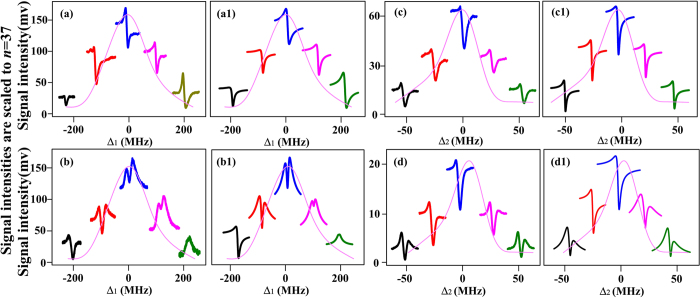
Dressed MWM processes by scanning Δ_4_. (**a-b**) The enhancement and suppression of FWM2 and SWM1 dressed by the SWM2 process with Δ_1_ growing at Δ_2_ = Δ_3_ = 0 for 37D and 54D, respectively. The Lorentzian profiles (curve constituted of the baseline of each signal) are the FWM2 and SWM1 signal versus Δ_1_ for 37D and 54D, respectively. (c-d) The enhanced and suppressed SWM1 by increasing the Δ_2_ with Δ_1_ = −Δ_3_ = 30 MHz for 37D and 54D, respectively. The Lorentzian profiles are the SWM1 signal versus Δ_2_ for 37D and 54D. (a1-d1) are the theoretical curves corresponding to (**a-d**) with ΔΦ′ = −*π*/6, respectively. The Rydberg-induced phase shift difference between 37D and 54D is about *π*/4. *N*_0_ = 1 × 10^12^ cm^−3^↔Ω_1_ = 2π × 54 MHz at 0.5 mW, Ω_2_ = 2π × 7.6 MHz at 200 mW, Ω_4_ = 2π × 116 MHz at 4 mW, Ω_3_ = 2π × 170 MHz at 5 mW, Ω_3_′ = 2π × 275 MHz at 13 mW.

Besides of the blockade dressed SWM process discussed above, one can further use the Rydberg MWM process to study the phase shift induced by the strong Rydberg-Rydberg interaction. Figures 4(a,b) show the induced enhancements and suppressions of FWM2 and SWM1 together with the SWM2 processes for 37D and 54D by varying Δ_1_ at Δ_2_ = Δ_3_ = 0, respectively. The peaks of the FWM2 and SWM1 signals versus Δ_1_ for 37D and 54D are shown by the Lorentzian profiles, which can be described by the one-photon term *γ*_1_ in Eqs. (4) and (5), respectively. Since the results are related to the changing of Δ_1_, they can be attributed to the dressing effects on the one-photon term *γ*_1_ as shown in Eqs. (4) and (5). The phase shift of ΔΦ′ on the dressing term |Ω_4_|^2^/*γ*_4_ is −*π*/6 (see Fig. 4(a1,b1)). The difference of the phase shifts induced by different cooperative nonlinear effect for the two principal quantum numbers can be obtained by comparing the corresponding modulated results at the same frequency detuning. In the current case, a phase shift difference of *π*/4 is introduced between 37D and 54D due to the *n*-dependent characteristic of cooperative nonlinearity.

Figure 4(c,d) are the enhanced and suppressed SWM1 for 37D and 54D at Δ_1_ = –Δ_3_ = 30 by altering Δ_2_, respectively. The Lorentzian profiles with linewidth of 60 MHz are the two-photon peaks of the SWM1 signal versus Δ_2_ for 37D and 54D, respectively, and related to the two-photon term *γ*_2_ in Eq. (5) (see Fig. 4(c1,d1)). Different from the former cases, we are now interested in the dressing effects on the two-photon term *γ*_2_ whereas the dressing effects on *γ*_1_ can be neglected. However, except for the increase of suppression in correspondingly modulated SWM1 signals of 37D and 54D, the dressed results are almost the same for both states due to the strong optical pumping. Therefore, the information of the phase difference in inverted-Y subsystem with optical pumping effect by changing Δ_2_ is not as obvious as in Y-type system by changing Δ_4_. Here, we have to mention that the central frequency shift of the Lorentzian profiles is observed due to the energy shift induced by different Rydberg-Rydberg interactions.

Finally, we characterize the blockaded enhancement and suppression results at Δ_1_ = –120 MHz in Fig. 4(a,b) as the functions of the probe field strength *P*_1_, the Rydberg state coupling field strength *P*_2_, and the coupling field strength *P*_4_ for three *n*D_5/2_ states. We expand Eqs. (3)~(6) as Taylor series based on the dressing fields. Taking Eq. (5) as an example, we have


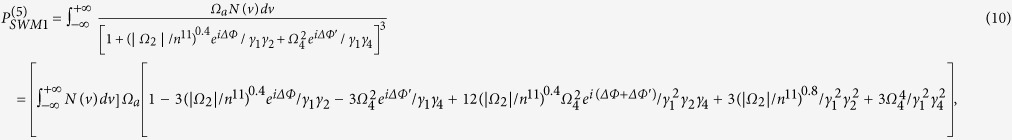


,where 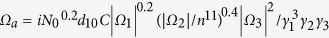. Therefore, the intensities of the enhanced peak, suppressed dip and background are related to the trems 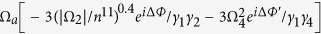, 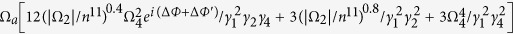, and 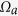, respectively. In addition, we have *I*∝*n*^−*3*^ according to Rydberg dressed MWM intensity *I*∝|Ω_*2*_|^2^∝|*d*_*ij*_|^2^ and *d*_*ij*_ ∝ *n*^(*−*3/2)^. Consequently, the Rydberg dressed signals for each principal quantum number *n* are scaled to *n* = 37 by the factor (n^*^/37^*^)^3^ accounting to the decrease in *d*_*ij*_ with increasing *n*. Here, n^*^ = *n*−*δ*, and *δ* = 1.35 is due to the quantum defect for nD_5/2_ state^27^.

**Figure 5:**
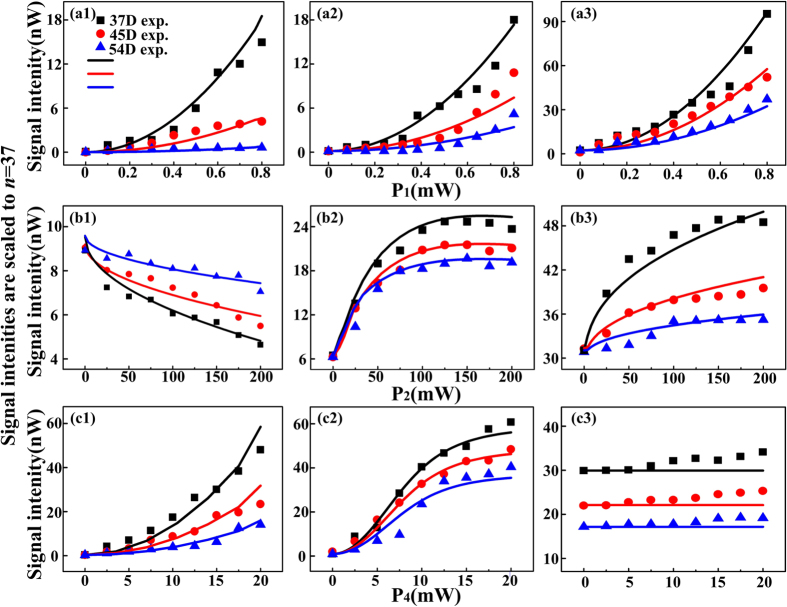
Power dependences (P_1_, P_2_ and P_4_ respectively) of the (a1, b1, c1) enhanced peaks, (a2, b2, c2) suppressed dips, and (a3, b3, c3) backgrounds, respectively, for three different *n*D_5/2_ states. The intensities of the Rydberg signals are scaled by (n^*^/37^*^)^3^ to account for the *n* dependence of the dipole matrix elements. *N*_0_ = 1 × 10^12^ cm^−3^. Δ_1_ = Δ_2_ = 0, Δ_3_ = 150 MHz. Ω_3_/2π = 170 MHz at 5 mW, Ω_3_′/2π = 275 MHz at 13 mW. (a1-a3) Ω_2_/2π = 7.6 MHz at 200 mW, Ω_4_/2π = 116 MHz at 4 mW. Ω_1_/2π grows from 0 to 68 MHz at 0.8 mW. (b1-b3) Ω_1_/2π = 54 MHz at 0.5 mW, Ω_4_/2π = 116 MHz. Ω_2_/2π grows from 0 to 7.6 MHz at 200 mW. (c1-c3) Ω_1_/2π = 54 MHz, Ω_2_/2π = 7.6 MHz, Ω_4_/2π grows from 0 to 259 MHz at 20 mW.

Figure 5(a) presents the ***E***_1_ power dependences of the (a1) enhanced peak, (a2) suppressed dip and (a3) background, respectively, for three *n*D_5/2_ states. The change of enhanced peak is mainly contributed by the enhanced FWM2 & SWM1 processes and the two-photon peak of SWM2. The trend of the suppressed dip can be understood as the saturating dressing-effect of ***E***_4_ at Δ_1_ + Δ_4_ = 0. The background evolution is due to the sum of FWM2 and SWM1 processes. Based on the evolutions of enhancement and suppression, we can draw the conclusion that the dressing asymmetry A_F_ increases with the strength of ***E***_1_. The saturating dressing-effect of ***E***_4_ means the phase shift is mainly caused by the existence of ***E***_2_ dressing and blockaded effect.

The cases of the ***E***_2_ power dependences for three *n*D_5/2_ states are shown in Fig. 5(b). First, we focus on the *P*_2_ dependence of the enhanced peak (see Fig. 5(b1)). At the low excitation intensity, the enhanced FWM2 signal and SWM2 signal contribute to the enhanced peak. As Ω_2_ increases, the enhanced SWM1 signal also makes the height of enhanced peak increase. However, the blockade term (|Ω_2_|/*n*^11^)^0.4^ makes the curve saturated at higher power level. Then, the descending part of the curve is due to the dressing effect of ***E***_2_ associated with its excitation blockade effect from |Ω_2_|/*n*^11^)^0.4^*e*^*i*ΔΦ^/γ_2_. Next, the power dependence of the suppressed dip can also reflect the blockade effect and the dressing effect of ***E***_2_ (see Fig. 5(b2)). Initially, the saturated dressing of ***E***_4_ on FWM2 signal at Δ_1_ + Δ_4_ = 0 and gradually increased SWM1 signal are the main factors. Then the curve becomes saturated due to the blockade term (|Ω_2_|/*n*^11^)^0.4^ at higher power level of ***E***_2_. As the power further increasing, the interaction between two dressing processes weakens the dressing results. Finally, one can obtain the direct blockade effect from the *P*_2_ power dependence of the background as shown in Fig. 5(b3). The background is consisted of FWM2 and SWM1 signals without the dressing effect of ***E***_4_. The saturation is due to the blockade effect, and the descending part is due to the combination of blockade effect and dressing effect of ***E***_2_. Given the above descriptions and analysis of peak and dip evolution corresponding to *P*_2_ strength dependence, one can deduce that the phase modulation as well as asymmetry can become more obvious by strengthening ***E***_2_. Meanwhile, we must note that the different principles for the increase of asymmetry are very corresponding to the three stages of power increase mentioned above. Lastly, ***E***_4_ power dependences in Fig. 5(c) just show the regular enhancement and suppression processes by ***E***_4_. The asymmetry changes are mainly aroused from the dressing effect of ***E***_4_.

## Discussion

The dressed suppression and enhancement of blockade MWM processes can reveal the change in nonlinear refractive index induced by cooperative atom-light interactions and corresponding dressing effects in Rydberg-EIT hot medium. On one hand, the observation of spatial shift and splitting effects of corresponding signals can visually advocate the dispersion property change of medium under blockaded effect. The transverse wave vector to explain the spatial effects is defined as


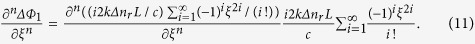


The first-order differential 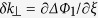 can describe spatial shift/splitting effects and the second-order differential 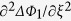 can explain the focusing/defocusing effects. On the other hand, the intensity modification of the enhanced and suppressed MWM signals obtained by scanning the dressing fields, which essentially control dark and bright states, can reflect the change in refractive index of a medium for a laser or MWM signals. Further, the cooperative nonlinearity induced phase modulation can be proportional to the refractive index change caused by Rydberg energy level shift. Consequently, we can quantificationally map the phase shift by cooperative nonlinear interaction onto suppression and enhancement of MWM processes involving in Rydberg states. With the dressing asymmetry A_F_ on the modulated results defined, A_F_∝(Δ_FWHM_/β)(α_1_ΔΦ + α_2_ΔΦ′) is established to depict the phase shift between dressing dark and bright states, where ΔΦ includes the phase shifts from both Rydberg dressing states and Rydberg excitation blockade and ΔΦ′ results from the orientations of induced dipole moments. The parameters α_1_ and α_2_ can be determined by experimental parameters such as the frequency detunings, Rabi frequencies, atom density and polarization states of laser fields.

## Methods

### Experimental setup

We use six light beams from three commercial external cavity diode lasers (ECDL) and one frequency-doubling laser system to couple a five-level X-type rubidium atomic system. The transition of *D*_*2*_ line is driven by weak laser beam ***E***_1_ stabilized to a temperature-controlled Fabry-Perot (FP) cavity. A pair of coupling beams ***E***_3_ and ***E***_3_′, also driving the transition of *D*_2_ line for different hyperfine configuration, are from another ECDL locked to the saturated absorption signal of rubidium atom. Beam ***E***_2_ driving the Rydberg excitation is a frequency-doubled laser with high stability. We get the needed 480 nm laser ***E***_2_ by the way of frequency doubling LD2 at ~960 nm with a periodically-poled KTP crystal in an external ring resonator to generate the second harmonic wave. The strong beam ***E***_2_ adding onto the beam ***E***_3_ (in the same direction), which counter-propagates with beam ***E***_1_, drives the highly-excited Rydberg transition. ***E***_4_ and ***E***_4_′ are from the same LD4. ***E***_4_ adds onto the beam ***E***_3_ by a cubic polarizing beam splitter (PBS) and ***E***_4_′ propagates with ***E***_3_′ symmetrically with respect to ***E***_2_. All beams are focused by two lenses (L1 and L2, respectively) with same focal length 500 mm before the cell and intersect at one point inside the cell. The 1 cm long rubidium cell is wrapped by *μ*-metal and heated by the heater tape. The optical depth (OD) is 70 for atom density of 1.0 × 10^12^ cm^−3^.

### Theoretical models for Δ*n*_*r*_ and ΔΦ_1_(U)

Nonlinear refractive index change is modeled by taking Δ*n*_r_ as the product of the slope of the dispersion (∂*n*_r_/∂*ω*_2_) and the energy level shift (Δ*ω*_2_) of the Rydberg state due to the Rydberg-Rydberg interaction. ∂*n*_r_/∂*ω*_2_ is derived from the real part of the complex susceptibility^28^ *χ* for stationary atoms and zero-coupling detuning as


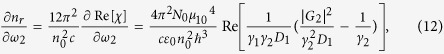


where *n*_0_ is the linear refractive index and *D*_*1*_* = *γ_*1*_* + |*Ω_*2*_*|/*γ_*2*_* + |*Ω_*4*_*|/*γ_*4*_. The energy level shift is


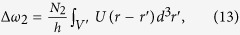


where *U*(*r* − *r*′) is the cooperative nonlinear interaction for Rydberg atoms at *n*D states; *N*_2_ is the density of excited Rydberg atoms. If we calculate the Rydberg excitation density via optical Bloch equation (OBE) by using the mean-field model^2^ and taking *N*_2_*V*_*d*_ = 1 & *V*_*d*_  ∝(*R*_*d*_)^3^ into account, the average Rydberg atom density *ρ*_e_ with considering of Doppler width Ω_D_ can be described as


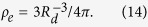


Here *R*_*d*_ is the radius of a Rydberg domain, which includes a single Rydberg atom and many ground-state atoms. By comparing with the non-blockade case, we find the following regulation as
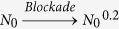
, 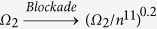. So the density of excited Rydberg atoms *N*_2_ is given as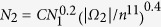, where *N*_1_ is the density of atoms at level |1〉. With the EIT effects and optical pumping effect taken into consideration, *N*_1_ is given by 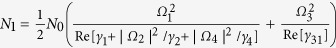, where *γ*_31_ = Γ_13_ + *i*Δ_3_; *C* is a constant mainly determined by the coefficient of Rydberg-Rydberg interaction and resulting from numerical integration outside the given sphere and the atom excitation efficiency between |0〉 and |1〉. Therefore, the change in refractive index can be defined as


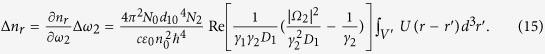


The induced phase modulation under the cooperative nonlinear interaction is


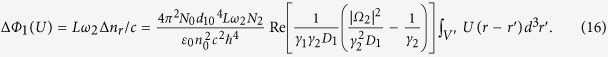


## Additional Information

**How to cite this article**: Zhang, Z. *et al*. Phase Modulation in Rydberg Dressed Multi-Wave Mixing processes. *Sci. Rep.* **5**, 10462; doi: 10.1038/srep10462 (2015).
